# Impact of the COVID-19 Pandemic on Pediatric Neuropsychiatric Disorders: A Retrospective Study at a Single Center for Child Neurology and Psychiatry

**DOI:** 10.3390/children13070926

**Published:** 2026-07-14

**Authors:** Giulia Spoto, Cecilia Spoto, Liliana Ruta, Gennaro Tartarisco, Roberta Bruschetta, Natalia Carolina Arminio, Caterina Sferro, Maria Spanò, Marilena Briguglio, Anna Cafeo, Greta Amore, Ambra Butera, Arianna Mancini, Arianna Currò, Maria Pia Lizio, Maria Ludovica Albertini, Carla Consoli, Graziana Ceraolo, Antonio Gennaro Nicotera, Gabriella Di Rosa

**Affiliations:** 1 Unit of Child Neurology and Psychiatry, Department of Biomedical Sciences, Dental Sciences & Morpho-Functional Imaging, University of Messina, 98125 Messina, Italy; giulia.spoto27@gmail.com (G.S.); gdirosa@unime.it (G.D.R.); 2 Faculty of Medicine and Surgery, Department of Human Pathology of the Adult and Developmental Age “Gaetano Barresi”, University of Messina, 98125 Messina, Italy; cecilia.spoto27@gmail.com; 3 Institute for Biomedical Research and Innovation (IRIB), National Research Council of Italy (CNR), 98100 Messina, Italy; liliana.ruta@irib.cnr.it (L.R.); gennaro.tartarisco@irib.cnr.it (G.T.); roberta.bruschetta@irib.cnr.it (R.B.); 4 Unit of Child Neurology and Psychiatry, Maternal-Infantile Department, University of Messina, 98125 Messina, Italy; natalia.arminio@polime.it (N.C.A.); caterina.sferro@polime.it (C.S.); maria.spano@polime.it (M.S.); marilena.briguglio@polime.it (M.B.); 5 Unit of Child Neurology and Psychiatry, Department of Human Pathology of the Adult and Developmental Age “Gaetano Barresi”, University of Messina, 98125 Messina, Italy; cafeoanna@gmail.com (A.C.); agreta18@gmail.com (G.A.); butera.ambra@gmail.com (A.B.); arianna.mancini89.am@gmail.com (A.M.); arianna.curro0511@gmail.com (A.C.); liziomariapia@gmail.com (M.P.L.); marialudovica.albertini@gmail.com (M.L.A.); carlaconsoli@hotmail.com (C.C.); graziana.c23@hotmail.it (G.C.); 6 Unit of Child Neurology and Psychiatry, Department of Chemical, Biological, Pharmaceutical & Environmental Science, University of Messina, 98125 Messina, Italy

**Keywords:** anorexia, anxiety, COVID-19, neuropsychiatric, eating disorders, somatic symptom disorders

## Abstract

**Background:** The COVID-19 pandemic and related containment measures profoundly affected child and adolescent mental health, with increasing evidence of a rise in psychiatric symptomatology and healthcare burden. This study aimed to evaluate temporal changes in neuropsychiatric presentations before, during, and after the COVID-19 pandemic in a pediatric clinical population. **Methods:** In this retrospective single-center study, 505 children and adolescents referred to a pediatric neuropsychiatry service were included and stratified into three periods: pre-pandemic (*n* = 112), pandemic (*n* = 231), and post-pandemic (*n* = 162). Clinical and healthcare-related variables were retrospectively collected, including reasons for referral, psychiatric diagnoses, premorbid conditions, and pharmacological treatments. Comparative analyses were performed to identify temporal variations in psychopathological profiles and clinical management. **Results:** A significant increase in psychiatric symptomatology emerged in the post-pandemic period compared with the pre-pandemic period. In particular, eating disorders, mood disturbances, self-harm, and somatic symptom presentations showed a marked increase over time, presenting as recurrent and associated phenotypes that peaked or consolidated after the acute emergency. The pandemic period was primarily characterized by a greater need for psychopharmacological intervention, specifically driven by the clinical need to manage acute behavioral crises. Overall, findings indicated a persistent increase in the complexity and severity of neuropsychiatric presentations beyond the acute phase of the pandemic. **Conclusions:** The COVID-19 pandemic was associated with substantial changes in pediatric neuropsychiatric presentations and treatment needs. The persistence of the increased psychopathological burden in the post-pandemic period highlights long-term mental health trajectories associated with the pandemic era. These emerging patterns of phenotypic complexity demonstrate that child and adolescent psychiatric services must transition from single-diagnosis models toward integrated care networks. Practically, strengthening community-based early intervention and creating rapid-access crisis support are essential to manage severe emotional dysregulation in the community and reduce acute psychiatric hospitalizations.

## 1. Introduction

The outbreak of the severe acute respiratory syndrome coronavirus 2 (SARS-CoV-2), responsible for the Coronavirus Disease 2019 (COVID-19), led the World Health Organization (WHO) to declare a global pandemic on 11 March 2020 [1]. Across the world, governments have introduced a range of social distancing strategies, and in Italy, a total national lockdown was declared to slow down the transmission of the virus. In Italy, this included the closure of schools, recreational facilities, and all shops, except those selling essential goods, and restrictions on traveling throughout the country [2]. A less restrictive period occurred starting in June, followed by a new lockdown in October 2020 [3]. The periods of social distancing and general closures persisted until April 2022, when the government declared the end of the emergency period in Italy [4].

An extensive body of literature has investigated the negative effects of the COVID-19 pandemic on the general population and, according to various studies, many people experienced negative emotional consequences during epidemics or disasters, as a result of the fear of contagion and the death of family members [5,6,7]. The COVID-19 pandemic has had disruptive repercussions on many dimensions of life for children and teens, significantly impacting many aspects of their mental health [8]. The global prevalence of anxiety, depression, and self-harm has risen significantly among the pediatric population, with an increase in symptom severity [9,10]. Autoaggressive and suicidal behaviors were more prevalent among adolescents compared to children, both before and during the COVID-19 pandemic; however, the prevalence of depression and anxiety increased during the pandemic, particularly among adolescents [11,12,13,14]. A study carried out in China on healthy children and adolescents found a 3% incidence of suicidal thoughts following the COVID-19 pandemic [15]. Adolescence was especially at risk because of their increased vulnerability to the lack of peer interactions during a critical window for neuropsychiatric development [16,17]. Recent data showed that during the COVID-19 pandemic, one in three adolescent girls and one in seven adolescent boys seriously considered suicide [18]. Moreover, it has been suggested that interpersonal difficulties may worsen the effect of isolation, and that people may respond differently to stressful events based on pre-existing psychopathological features, which may increase susceptibility to the emotional consequences of any disaster-related trauma [19,20,21]. This was particularly evident in the months following the lockdowns, with a marked increase observed after the second period of restrictions. In fact, while severe conditions such as agitation, psychotic symptoms, and suicidality (suicidal ideation/self-harm/suicide attempts) were the primary reasons for psychiatric referrals during the first lockdown, anxiety and depressive disorders underwent a significant increase in the following months [22]. Suicide rates among 13- to 17-year-olds escalated significantly from July to December 2020 and a 51% rise was registered in emergency room visits with suspected suicide attempts among girls aged 12–17 in early 2021 compared to the same period in 2019 [18,23]. In Italy, Pontillo and colleagues showed a significant increase in mood disorders among patients admitted during the second lockdown compared to the same period of the previous year and a higher rate of suicidal ideation among children and adolescents hospitalized during the quarantine period [24]. Additionally, a study conducted in China by Zheng and colleagues during the late phase of the pandemic (November 2021 to January 2022) found that 43.27% of the patients with a prior diagnosis of mood disorder reported non-suicidal self-harm episodes within the previous month [25].

Beyond explicit psychiatric disorders, the pandemic-related trauma significantly influenced the somatic expression of distress in the pediatric population. In this context, headaches represent one of the most prevalent forms of pain in childhood and adolescence, with psychological stress widely recognized as a primary trigger [26,27]. Loneliness and social isolation have been consistently linked to heightened risks of adverse somatic health outcomes, including the onset or exacerbation of headache and physical pain in children and adolescents [28,29,30]. Because psychological distress and headache frequently co-occur and share common underlying risk factors, they represent closely interconnected conditions rather than independent phenomena [31]. Therefore, evaluating tension-type headache alongside overt behavioral and emotional symptoms is crucial to capture the full spectrum of pediatric responses to the pandemic, including those patients who channel psychological trauma into somatic rather than purely psychiatric pathways.

While the broad psychological toll of the COVID-19 pandemic on youth is well-documented, the current literature often evaluates psychiatric symptoms or reasons for admission as isolated clinical entities. This leaves a critical gap in understanding how specific neuropsychiatric symptoms acutely overlapped and collapsed into complex clinical presentations during the acute and post-acute phases of the crisis. Furthermore, data regarding how these intertwined clinical emergencies directly translated into inpatient acute pharmacological strategies within child and adolescent neuropsychiatry settings remain scarce [32,33].

To address this gap, this study primarily aims to examine the clinical presentations and specific symptom clustering of children and adolescents acutely admitted to our Child and Adolescent Neuropsychiatry Unit across three distinct pandemic phases (Pre-COVID-19, COVID-19, and Post-COVID-19). As secondary objectives, we aimed to: (i) analyze changes in the patterns of psychiatric diagnoses and admission reasons, and (ii) evaluate modifications in the pharmacological treatment strategies introduced during hospitalization. By clarifying these complex clinical presentations—which we define here as novel, multifaceted combinations of overlapping neuropsychiatric symptoms rather than distinct, new diagnostic entities—this study aims to provide insights into the evolving emergency neuropsychiatric needs in the aftermath of global health crises.

## 2. Methods

### 2.1. Patients and Study Design

Patients were retrospectively recruited from the Unit of Child Neurology and Psychiatry at the University Hospital “G. Martino” in Messina, Italy, between December 2020 and April 2023.

Patient selection was conducted by two researchers (G.S., G.A.) based on discharge diagnoses formulated by senior child and adolescent neuropsychiatrists (N.C.A., C.S., M.S., M.B., A.G.N., G.D.). Given the retrospective design, researchers were not blinded to the pandemic time periods.

The population was selected according to the following inclusion criteria:

(1) age between 3 and 17 years;
(2) hospitalization required due to psychiatric symptoms, with or without somatic complaints;
(3) a psychiatric diagnosis according to the Diagnostic and Statistical Manual of Mental Disorders, Fifth Edition [34] or a diagnosis of “Tension-type Headache” according to the International Classification of Headache Disorders-3 [35].

Exclusion criteria were established as follows:

(1) patients aged outside the 3–17 range;
(2) admissions lasting less than 48 h (e.g., brief observation in the emergency department without subsequent ward hospitalization);
(3) admissions for purely neurological conditions lacking any concurrent acute psychiatric, emotional, or behavioral presentation (e.g., epilepsy, acute neurological deficits, or acute neuromyelitis optica);
(4) medical records with substantial illegibility or missing data regarding the primary diagnosis or reason for admission.

The study population was chronologically divided into three periods: (1) Pre-COVID-19 (March 2019 to February 2020; 12 months); (2) COVID-19 (December 2020 to March 2022; 16 months); and (3) Post-COVID-19 (April 2022 to April 2023; 13 months). The transition phase from March to November 2020 was excluded due to both institutional and psychological factors. Logistically, during the first and second regional lockdown waves, inpatient admissions were strictly restricted to non-deferrable neuropsychiatric emergencies, while spontaneous presentations were heavily altered by public fear of infection. Psychologically, while the first wave was met with initial community resilience and temporary coping strategies, the second lockdown in late 2020 marked a definitive shift toward social exhaustion, collective demoralization, and chronic distress. By centering the ‘COVID-19 period’ from December 2020 onwards, we aimed to precisely capture the long-term, stable changes in pediatric phenotypes that occurred after prolonged pandemic stress, avoiding the highly unstable clinical and administrative data of the initial waves.

### 2.2. Data Collection and Clinical Definitions

The data collected included patient age, reason for admission, length of hospital stay, urgency of hospitalization (planned for follow-up, urgent due to symptom severity, or through the emergency unit), and discharge diagnosis.

Hospital admission reasons were categorized as ‘agitation,’ ‘suicidality,’ ‘self-harm,’ ‘mood disturbance,’ ‘eating disorder,’ ‘anxiety,’ ‘headache,’ and ‘neurological-like symptoms’. The category of ‘neurological-like symptoms’ was operationally defined to include functional gait disturbances, dizziness/vertigo, psychogenic non-epileptic seizures (PNES), and psychogenic vomiting. To exclude primary organic etiologies, a standardized diagnostic protocol was systematically applied to all patients in this category. This baseline workup comprised a formal neurological examination performed by senior child neurologists (N.C.A., C.S., M.S., M.B., A.G.N., G.D.), routine blood chemistry panels, standard electroencephalography (EEG), and comprehensive psychological evaluations. Advanced diagnostic investigations were reserved for specific clinical indications: specialized consultations (e.g., gastroenterology for persistent vomiting) and neuroimaging (brain/spinal Magnetic Resonance Imaging) were performed on an as-needed basis to definitively rule out structural or systemic neurological pathologies before confirming a functional/psychosomatic origin.

Diagnoses were categorized as ‘Neurodevelopmental disorders’, ‘Psychotic disorders’, ‘Mood disorders’ (including Bipolar and Depressive disorders), ‘Anxiety disorders’ (including Obsessive–Compulsive Disorder), ‘Trauma- and Stressor-Related disorders’, ‘Somatic Symptoms disorders’, ‘Eating disorders’, ‘Externalizing disorders’, and ‘Tension-type Headache’. The rationale for retaining tension-type headache relies on its established nature in developmental ages as a key somatic manifestation of internalized psychological distress. Capturing both behavioral and somatic/functional presentations allowed for a comprehensive mapping of the full neuropsychiatric spectrum managed by the unit during the pandemic phases. Tension-type headache was treated as a dichotomous clinical variable within the overall cohort to evaluate its correlation patterns with psychiatric phenotypes (such as anxiety and mood disorders) across the three pandemic phases.

We also collected data on medical history, including premorbid conditions and family history for neuropsychiatric pathologies, eating habits, sleep disturbances, and pharmacological treatment before and after the hospitalization.

All clinical data were systematically extracted from the standardized institutional medical charts by a separate team of researchers (C.S.1, A.B., A.M., A.C.1). Any doubts or ambiguities regarding the data extraction or the classification of specific psychiatric presentations were discussed and resolved through consensus with the senior specialists. Inter-rater reliability was not formally assessed. Crucially, reasons for admission were not treated as mutually exclusive categories but as independent dichotomous variables (0 = absent, 1 = present). If a patient presented with multiple concurrent clinical symptoms, all relevant presentations were systematically scored as present, allowing for the subsequent evaluation of symptom co-occurrence. Regarding data completeness, core variables such as age, sex, primary admission symptoms, and final psychiatric diagnoses were fully documented across the database. Missing data encountered during the retrospective chart review—primarily concerning family psychiatric history, specific socio-demographic details (education, parental status), and baseline physiological habits (sleep and eating patterns)—were systematically coded (e.g., using ‘999′ placeholders) and addressed using an available-case analysis approach. Consequently, cases with missing values in secondary variables were excluded only from the specific analyses involving those parameters, preserving the maximum available sample size for each comparison. No statistical imputations were applied.

### 2.3. Statistical Analysis

Continuous variables, including patient age and length of hospital stay, were first evaluated for normality. Because these parameters displayed a non-normal distribution, a non-parametric approach was utilized. Specifically, global comparisons across the three pandemic groups were performed using the Kruskal–Wallis H test.

For categorical data and frequency distributions (including gender, admission pathways, clinical phenotypes, and pharmacological treatment patterns), statistical comparisons across the three study periods were conducted using Pearson’s Chi-square (χ^2^) test. To explore the relationships and overlapping presentation patterns among reasons for hospital admission within each separate study period, bivariate correlation matrices were computed. Given that all admission-reason variables were coded as dichotomous (0/1), the mathematical approach utilized Pearson’s equation, which under binary conditions simplifies to and functions exactly as the Phi (ϕ) coefficient, providing a standard measure of association for binary pairs. Statistical significance for each correlation was determined via two-tailed tests based on the specific sample size of each subpopulation. Regarding the interpretation of correlation strength, coefficients between 0.10 and 0.29 were considered weak, between 0.30 and 0.49 moderate, and ≥0.50 strong. To ensure full methodological transparency, a comprehensive summary presenting both the exact correlation coefficients and their respective exact *p*-values across all three periods has been added to the Supplementary Material (Supplementary Tables S1–S3). Given the exploratory nature of these bivariate correlation matrices, which were conducted to identify shifting clinical patterns and generate hypotheses regarding symptom co-occurrence across different pandemic phases rather than to test a single a priori confirmatory hypothesis, no formal alpha-taming adjustments for multiple comparisons (such as Bonferroni or False Discovery Rate) were applied. Consequently, borderline significant correlations (*p* ≈ 0.04) were interpreted with marked caution throughout the text, while robust associations (*p* < 0.001) were highlighted as structurally stable. All statistical testing was two-tailed, and the threshold for significance was set at *p* < 0.05.

The statistical analysis was performed using SPSS software version 25.0 (IBM SPSS Statistics, Chicago, IL, USA).

## 3. Results

A total of 505 patients were included in this study, comprising 252 males (49.9%) and 253 females (50.1%). The COVID-19, post-COVID-19, and pre-COVID-19 groups encompassed 231, 162, and 112 patients, respectively. Demographics of the patients and hospitalization data are summarized in Table 1.

No significant differences were found among the three groups regarding male/female ratios and age of the patients at admission. During the COVID-19 period, 27 patients required more than one hospitalization, with progressively longer durations of stay; in the post-COVID-19 group, 10 patients were readmitted at our unit, while during the pre-COVID-19 period 8 patients required multiple hospitalizations. No significant differences were found among multiple hospitalizations in the three groups.

Admission through the emergency unit was significantly higher during the COVID-19 period compared to the pre-COVID-19 and post-COVID-19 periods (*p* = 0.01 and *p* < 0.0001, respectively).

Urgent admission due to the worsening of symptoms was significantly higher during the post-COVID-19 period compared to the pre-COVID-19 and COVID-19 periods (*p* = 0.018 and *p* = 0.025, respectively).

**Figure 1:**
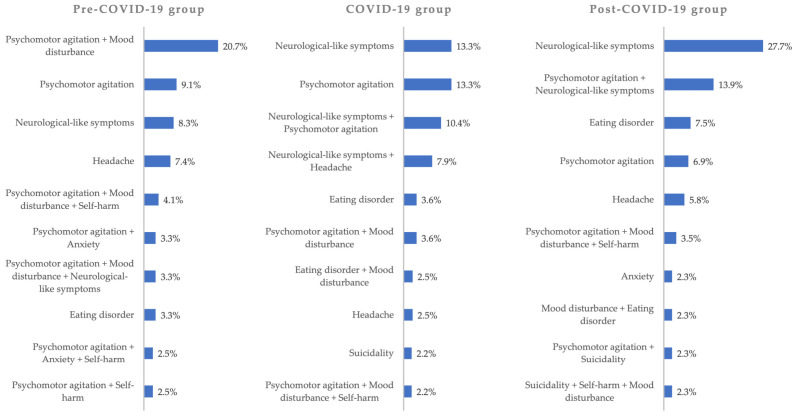
The graphs show the 10 most common reasons for admission during the pre-COVID-19, COVID-19, and post-COVID-19 periods, ordered by frequency.

To better characterize the clinical profile of the patients, we evaluated the symptoms for which they required hospitalization, either individually or in combination. The most frequent reason for admission in the pre-COVID-19 group was the combination of agitation associated with mood disturbances (20.7%), while in the COVID-19 and post-COVID-19 groups the patients were primarily admitted for neurological-like symptoms (13.3% and 27.7%, respectively). Figure 1 summarizes the main reasons for admission during the three periods.

**Figure 2:**
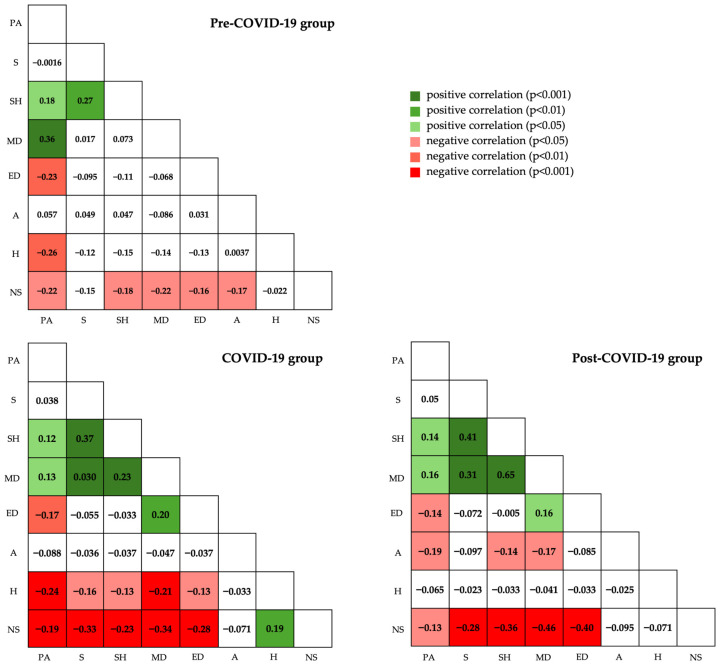
Correlation matrices of reasons for admission in the pre-COVID-19, COVID-19, and post-COVID-19 groups. The strength and direction of associations are expressed via Phi (ϕ) correlation coefficients, ranging from −1.0 to +1.0. Positive correlations are displayed in green shades, while negative correlations are shown in red shades, with darker tones indicating stronger associations. Comparing the matrices, the shift in clinical presentation of our patients is observable. A positive association among self-harm, suicidality, and mood disorders emerged during the COVID-19 period and strengthened in the post-COVID-19 period. On the contrary, a negative association among neurological-like symptoms and psychiatric manifestations such as psychomotor agitation, suicidality, self-harm, mood disorders, and eating disorders had its peak in the COVID-19 period and slightly decreased in the post-COVID-19 group. Legend: A: anxiety; ED: eating disorder; H: headache; MD: mood disturbance; NS: neurological-like symptoms; PA: psychomotor agitation; S: suicidality; SH: self-harm.

To assess the extent of association between these symptoms, we subsequently analyzed them using correlation matrices. As visually summarized in Figure 2, several clinical patterns emerged across the three periods; for the complete correlation matrices containing exact coefficient values and exact *p*-values, see Tables S1–S3 in the Supplementary Material.

Premorbid conditions revealed a significantly higher prevalence of eating disorders in the post-COVID-19 period compared to the pre-COVID-19 period (*p* = 0.04). In addition, a previous diagnosis of Externalizing Disorder was significantly higher in the post-COVID-19 group than in the COVID-19 group (*p* = 0.03).

There was no significant difference in the distribution of diagnoses between the pre-COVID-19 and COVID-19 periods, nor between the COVID-19 and post-COVID-19 periods. However, a significant difference was found between the post-COVID-19 and the pre-COVID-19 periods (*p* = 0.021). Particularly, the number of patients with a diagnosis of Neurodevelopmental disorders was statistically higher in the post-COVID-19 group than in the pre-COVID-19 and COVID-19 groups (*p* = 0.005). On the contrary, a diagnosis of Externalizing disorders was statistically higher in the pre-COVID-19 period in comparison to the COVID-19 and post-COVID-19 periods (*p* < 0.001). Moreover, the Psychotic disorders were significantly higher during the COVID-19 period than in the post-COVID-19 period (*p* = 0.024).

Traumatic events were reported by 20.7% of pre-COVID-19 patients, 30.3% of COVID-19 patients, and 18.5% of the post-COVID-19 group, with a significantly higher percentage observed in the COVID-19 group compared to the post-COVID-19 group (*p* = 0.008).

A family history of neuropsychiatric conditions was described in 82% of the patients in the pre-COVID-19 group, in 82.5% of the patients in the COVID-19 group, and in 71.3% of the patients in the post-COVID-19 group, which was significantly lower in the last period than in the other two periods (*p* = 0.044 and *p* = 0.008, respectively).

In the COVID-19 group, 39.2% of the patients were already on pharmacological treatment (28.1% on antipsychotics; 15.1% on mood stabilizers; 11.5% on benzodiazepines; 8.4% on antidepressants; 0.4% on psychostimulants). During hospitalization, 47.5% of the sample started a pharmacological therapy, 5.8% increased their dosage, and 11.5% switched to a different pharmacological class. The most used drugs during the COVID-19 period were antipsychotics (47.5%), followed by mood stabilizers (30.1%), benzodiazepines (29.5%), antidepressants (10.1%), and psychostimulants (2.9%).

In the post-COVID-19 period, 37.6% of the admitted patients were on pharmacological treatment (28.9% on antipsychotics; 19.7% on mood stabilizers; 14.5% on benzodiazepines; 5.2% on antidepressants; 2.3% on psychostimulants). Within this group, 47.4% started a pharmacological treatment, 5.8% increased their dosage, and 16.2% switched to a different pharmacological class. Antipsychotics were the most used drugs (47.4%), followed by benzodiazepines (34.1%), mood stabilizers (29.5%), antidepressants (6.9%), and psychostimulants (3.5%).

In the pre-COVID-19 period, 28.1% of the admitted patients were on pharmacological treatment (20.7% on antipsychotics; 13.2% on mood stabilizers; 13.2% on benzodiazepines; 1.7% on antidepressants). Pharmacological treatment was started in 33.1% of the patients, 9.1% required an increased dose of treatment and 5.8% needed a switch of pharmacological class. The most used drugs were the antipsychotics (37.2%), followed by mood stabilizers (20.7%), benzodiazepines (19%), antidepressants (12.4%), and psychostimulants (1.7%).

The initiation of pharmacological therapy was significantly higher in the COVID-19 and post-COVID-19 groups compared to the pre-COVID-19 period (*p* = 0.012 and *p* = 0.019, respectively). A statistically significant reduction in the use of antidepressants was observed between the pre-COVID-19 period and the post-COVID-19 period (*p* = 0.045). A significant increase in benzodiazepine use was observed during the COVID-19 and post-COVID-19 periods compared to the pre-COVID-19 period (*p* = 0.038 and *p* = 0.006, respectively).

A comprehensive summary of psychopharmacological pre-admission treatments, in-hospital management, and most frequently used drug classes across the three study periods is provided in Table 2.

When examining the prescriptions at discharge for the high-risk subgroup of patients admitted with mood instability or suicidality across the distinct study phases (61 in pre-COVID-19, 82 in COVID-19, and 44 in post-COVID-19 groups), a clear therapeutic shift emerged. The initiation of antidepressants remained consistently low across all periods (9.8% in pre-COVID-19, 13.4% in COVID-19, and 9.1% in post-COVID-19). Conversely, to counter the prominent behavioral activation and psychomotor agitation complicating these acute phases, a substantial increase was observed in the prescription of atypical antipsychotics—which rose from 42.6% in pre-COVID-19 to 68.3% during the COVID-19 peak, reaching 72.7% in the post-COVID-19 phase—and mood stabilizers, which scaled from 31.1% in pre-COVID to 46.3% in COVID-19 and 68.2% in post-COVID-19. From a clinical standpoint, this trend is visually reflected by the presence of concurrent psychomotor agitation, where the rate of antidepressant introduction was lower compared to non-agitated peers (8.8% vs. 16.4%), in favor of atypical antipsychotics and mood stabilizers as first-line tools for immediate behavioral stabilization.

## 4. Discussion

The COVID-19 pandemic significantly affected the mental health of children and adolescents, leading to a marked increase in psychiatric disorders. This increase was not only evident in individuals with a previous diagnosis of a psychiatric disorder but also in those with a previously silent psychiatric history [4,8,36]. Globally, during the COVID-19 pandemic, emergency room admissions were primarily due to anxiety, depression, psychomotor agitation, and suicidal attempts and ideation [37,38,39,40]. In Italy, however, healthcare service admissions increased during the second lockdown, possibly due to fear of contagion [22]. Consistent with these data, in our sample, admission through the emergency unit was significantly higher during the COVID-19 period than during the other periods. While this finding potentially reflects a higher acute psychological burden, it must also be interpreted in light of healthcare access modifications. The variable accessibility and intermittent disruption of outpatient and community mental health services across the different pandemic phases likely shifted many pediatric patients toward emergency pathways as the most reliable point of care. In addition, we observed a shift in the psychiatric phenotype compared to the pre-COVID-19 period (see Figure 2), highlighting the need to understand these changes for effective mental health support.

Agitation, particularly when associated with mood disorders and anxiety, has always been considered one of the primary reasons for admission to emergency departments and a common presentation of psychiatric disorders in children and adolescents [41,42,43]. Accordingly, psychomotor agitation was a significant factor in our pre-COVID-19 admissions and the main reason for a psychiatric hospitalization in our unit. However, in our cohort, the association between agitation and mood disturbances decreased during the COVID-19 period, and although it increased during the post-COVID-19 period, it remained lower than in pre-COVID-19 patients. In contrast, mood disorders showed a rising trend in their association with self-harm and suicidality. Crucially, our correlation analysis revealed a remarkably strong post-pandemic coupling between Mood Disorders and Self-Harm (ϕ = 0.65, *p* < 0.001), suggesting a progressive phenomenological convergence where acute affective dysregulation and direct physical self-injury increasingly co-occurred as a tightly integrated clinical presentation at admission. Previous studies suggested that depression, combined with pandemic-induced loneliness, exacerbates the risk of developing suicidal ideation, particularly in individuals with pre-existing mental health conditions and mood disorders [8,19,20,21,44]. A study conducted by Zheng and colleagues evaluated the incidence and characteristics of self-harm in patients diagnosed with mood disorders during the late phase of the pandemic (November 2021 to January 2022). The results showed that 43.27% of the patients (aged between 14 and 44 years, with a prior diagnosis of mood disorder and no accompanying psychotic symptoms) reported non-suicidal self-harm episodes within the previous month, and that this practice was quite prevalent among students and adolescents with comorbid Obsessive–Compulsive Disorder symptoms [25]. In Italy, Pontillo and colleagues analyzed psychopathological disorders and self-injurious behaviors in 377 patients aged between 6 and 18 years, showing a significant increase in mood disorders among patients admitted during the quarantine period (second lockdown) compared to the same period of the previous year. The authors also found an increase in suicidal ideation among children and adolescents hospitalized during the quarantine period (i.e., 40.4% vs. 29.6%) [24]. The impact of the pandemic on the increased risk of suicidality, particularly in patients with a prior diagnosis of mood disorders, has also been confirmed by studies on adult patients [45,46,47]. Although the association between self-harm and suicidality has been previously described in the literature, estimates of COVID-19-related changes in severe mental distress indicators, including suicide attempts, self-harm, and suicidal thoughts, have varied across the literature [10,16]. Before the COVID-19 pandemic, suicidal ideation and self-harm were extremely common: one in seven adolescents, aged 12 to 16, reported having considered suicide, and one in six adolescents, aged 13 to 18, reported engaging in self-harm behavior [48]. However, in a retrospective cohort study, Ougrin and colleagues found that the percentage of young people presenting self-harm increased from 50% in 2019 to 57% in 2020 [10]. An Italian study conducted by Beghi and colleagues on emergency room admissions of pediatric and adult patients for psychiatric evaluation after the (first) lockdown showed that agitation, psychotic symptoms, and suicidality were the prominent reasons for psychiatric referral during the COVID-19 pandemic [22]. Accordingly, we also note a steadily increasing correlation between self-harm and suicidality in our cohort, rising from a correlation coefficient (ϕ) of 0.27 in the pre-COVID-19 period to 0.37 during the COVID-19 phase, and peaking at 0.41 in the post-COVID-19 period. This post-pandemic consolidation stands in contrast to weaker symptom overlaps that emerged during the pandemic peak. Specifically, the association between agitation and self-harm decreased to a correlation coefficient of 0.12 during the COVID-19 period (second lockdown), compared to 0.14 in the post-COVID-19 period and 0.18 in the pre-COVID-19 era. While statistically significant due to the statistical power of the larger COVID-19 subgroup sample size (*p* = 0.046), this low coefficient indicates an occasional or highly heterogeneous clinical overlap rather than a systematic syndromic trend. The long-term effects of the pandemic on this internalizing spectrum became even more prominent following the lifting of lockdowns and associated restrictions. Indeed, in our cohort, the correlations between anxiety and mood disturbances, self-harm, and psychomotor agitation only reached full statistical significance within the post-COVID-19 group. It is well-established that anxiety disorders can increase the risk of developing major depressive disorder and that these two combined disorders can heighten suicidal ideation and suicide attempts [49,50]. Moreover, loneliness is considered a significant risk factor for severe psychopathological symptoms in the pediatric population and some studies suggested that the pandemic might have led to post-traumatic stress in children and adolescents, explaining the increasing correlation exhibited by the post-COVID-19 group in our population [16,22,51,52].

Parallel to this intensification of internalizing psychiatric symptoms, the emotional distress provoked by the pandemic frequently manifested through physical and somatic pathways. During the COVID-19 period, our patients also showed significant mild correlations between mood disturbances and eating disorders (ϕ = 0.20), which remained statistically significant in the post-COVID-19 group (ϕ = 0.16). We also noticed an increase in the frequency of eating disorders among the reasons for hospitalization, rising from 3.3% in the pre-COVID-19 group to 3.6% in the COVID-19 period and 7.5% in the post-COVID-19 group. Although we hypothesize that pandemic-related stress and the associated psychosocial burden may have played a key role, a causal link cannot be definitively established, and this upward trend should be interpreted as a significant temporal association rather than a direct consequence. It has been suggested that the COVID-19 lockdown may have triggered the onset of eating disorders or exacerbated pre-existing symptoms, including binge eating, restrictive dieting, and body image concerns [53,54]. Monteleone and colleagues documented a worsening of both general and eating psychopathology during lockdown periods, with anxiety symptoms increased not only during the lockdown (compared to the non-pandemic period) but also throughout the re-opening phase [55]. Moreover, an increase in hospitalizations as a treatment for eating disorders has been registered among children and adolescents [56,57]. In the systematic review conducted by Devoe and colleagues, an increase of more than 80% in pediatric hospitalization was observed during the COVID-19 pandemic, indicating that eating disorder symptoms, anxiety, and depression symptoms were elevated. Indeed, anxiety and depression symptoms were considered strong predictors of eating disorders in children and adolescents [54,58].

Another peculiar aspect of the shift in the phenotype presentation of our cohort was that the primary reason for hospitalization during the COVID-19 and post-COVID-19 periods was “neurological-like symptoms”, which included a range of organic-like symptoms that appeared as part of a neurological disorder but were later confirmed as being of psychosomatic origin. Notably, these symptoms showed significant negative correlations with most other reasons for hospitalization, demonstrating an increasing trend with mood disturbances and self-harm during the COVID-19 period and even more so in the post-COVID-19 period. Furthermore, the association with suicidality, eating disorders, and headache peaked during the COVID-19 period, with the first two lowering but remaining statistically significant even in the post-COVID-19 period. These negative correlations suggest that physical symptoms may underlie psychological distress that cannot be expressed otherwise [59].

Somatic symptoms and related disorders are frequent in the pediatric population, particularly during early adolescence [60]. Apart from interpersonal trauma, increased somatization in children has been observed following other traumatic environmental events, such as earthquakes and terrorist attacks [61,62]. Similarly, during the COVID-19 pandemic, there was an increase in the rate of emergency department admissions for somatic symptom disorders compared to the pre-pandemic year. In fact, Turco and colleagues found that gastrointestinal symptoms, rather than pain or pseudo-neurological symptoms, were more frequently presented in the pandemic group compared to the pre-pandemic group [63]. However, new diagnoses of functional neurological disorders, such as functional tic-like behaviour and PNES, were also reported in patients with psychiatric comorbidities [64,65,66]. Shahini and colleagues proved a significantly higher risk for somatic symptoms in adult patients with mood disorders [59]. Conversely, there is a notable paucity of literature regarding the impact of the COVID-19 pandemic on somatic symptom disorders in children, as well as on the clinical and management aspects of these conditions during this period. Pruccoli and colleagues investigated the variability of clinical features reported by children with somatic symptom disorders in the pre- and post-pandemic periods. The authors found a statistically significant increase in symptoms such as headache, fever, and asthenia during the pandemic compared to the previous period. Interestingly, these symptoms are similar to those of COVID-19, but SARS-CoV-2 infection was excluded through molecular nasopharyngeal swabs [67]. In addition, an interesting Italian study conducted by Conti and colleagues examined emotional and behavioral changes during the COVID-19 lockdown in a sample of 141 children with neuropsychiatric disorders, using the Child Behavior Checklist completed by parents as an assessment measure. The authors found a significant worsening in the Syndrome Scale Score, particularly in the Somatic Complaints and DSM-Oriented Anxiety Scale, in the preschool subgroup, and a significant worsening in the Syndrome Scale Score (Thought problems), Obsessive scale, and Post-Traumatic Stress Disorder scale in the schooler subgroup [68]. Similar to somatic symptoms, in our cohort, headache showed significant negative correlations with mood disturbances, self-harm, suicidality, and eating disorders. However, this significance disappeared in the post-COVID-19 period. The COVID-19 pandemic triggered both individual and communal psychological reactions, as well as a source of stress, which may have had an impact on children with primary headache illnesses [69,70]. Papetti and colleagues conducted a multicenter study to investigate how children and adolescents with primary headache disorders were affected by stress and lifestyle changes secondary to the COVID-19 lockdown. The authors found that general anxiety and depressed mood were associated with increasing headache trends, as well as frequency and intensity of attacks [71]. In fact, several studies have demonstrated that anxiety may be a triggering component in the development of headaches [72,73,74]. Furthermore, evidence suggests that some children may be less able to cope with daily life stressors, resulting in a higher frequency and intensity of headaches [75].

Another interesting finding in our cohort is the significantly lower percentage of family history of neuropsychiatric conditions in the post-COVID-19 group compared to earlier periods. This suggests that the traumatic impact of the COVID-19 pandemic may have potentially affected not only patients with a genetic predisposition but also those without a family history of such conditions [4,8,36,76].

We found no significant difference in the distribution of diagnoses between the pre-COVID-19 and COVID-19 periods and between the COVID-19 and post-COVID-19 periods; on the contrary, a significant difference was found between the post-COVID-19 and pre-COVID-19 periods, suggesting a slow modification of the clinical presentation shown by the patients needing hospitalization. Particularly, the number of patients with a diagnosis of Neurodevelopmental disorders was statistically higher in the post-COVID-19 group compared to the pre-COVID-19 and COVID-19 groups. It may be hypothesized that this trend reflects a combination of organizational and clinical factors. On one hand, this trend could potentially reflect organizational delays; the reduced availability of outpatient clinics during the acute pandemic phases may have deferred routine assessments, leading to a subsequent accumulation of newly diagnosed cases once services normalized. On the other hand, during the lockdown, it was not possible for children affected by these disorders, such as Autism Spectrum Disorder, Attention Deficit/Hyperactivity Disorder (ADHD), cerebral palsy, or developmental delay, to attend their regular therapy sessions, which could have led to increases in self-harm and reactive behaviors, anxiety and depression [77,78,79,80]. On the contrary, a diagnosis of Externalizing disorders was statistically higher in the pre-COVID-19 period in comparison to the COVID-19 and post-COVID-19 periods. As a general trend, it seems that the pandemic has caused an increase in externalizing disorders in individuals with pre-existing behavioral conditions [81]. However, some patients showing social and academic difficulties may have experienced less anxiety and mood enhancement during school closure periods, explaining the lesser need for hospitalization during these periods [82]. In addition, an increase in diagnosis of Psychotic disorders was recorded only in the COVID-19 group. It has been suggested that loneliness and psychosocial stress caused by social distancing could potentially contribute to psychosis onset [83,84]. However, an implication of the SARS-CoV-2 infection in the onset of psychotic symptoms has also been reported in the literature, reinforcing the hypothesis of the role of viral infections in the onset of schizophrenia and other neuropsychiatric conditions [19,85,86,87]. Unfortunately, individual data on SARS-CoV-2 infection status were not systematically available in this retrospective cohort. Therefore, our study cannot distinguish between the potential direct viral/immunological mechanisms triggering psychotic symptoms and the broader, profound psychosocial stressors and healthcare-related disruptions associated with the pandemic context.

Finally, we observed a statistically significant increase in the initiation of pharmacological therapy in the COVID-19 and post-COVID-19 groups compared to the pre-COVID-19 period. Specifically, there was a significant reduction in the use of antidepressants between the pre-COVID-19 and post-COVID-19 periods. This shift reflects a change in the clinical phenotype of patients admitted during the pandemic, who more frequently presented with complex profiles characterized by acute psychomotor agitation and severe emotional dysregulation rather than isolated depressive symptoms. Although the literature reported inconclusive results in the pediatric population, recent data showed that antidepressants may present slightly increased odds of suicide-related outcomes [88]. Consistently, in our cohort, antidepressant introduction was lower in patients with active psychomotor agitation compared to non-agitated peers (8.8% vs. 16.4%), as clinicians prioritized atypical antipsychotics and mood stabilizers for safe behavioral stabilization. Additionally, a statistically significant increase in the use of benzodiazepines was recorded in our cohort during the COVID-19 and post-COVID-19 periods in comparison to the pre-COVID-19 period. A recent meta-analysis confirmed that benzodiazepines are significantly more effective than antidepressants in treating the somatic symptoms of generalized anxiety disorder [89]. Therefore, their use as adjunctive therapy to antipsychotics and mood stabilizers was the preferable, safety-driven treatment to manage acute behavioral activation in our cohort.

From a clinical standpoint, the observed variations in symptom clustering and psychopharmacological patterns reflect an evolving clinical reality where acute admissions are increasingly driven by urgent behavioral crises rather than isolated distress. The significant post-pandemic coupling between mood disturbances and self-harm highlights that emotional dysregulation in youth now carries a heightened risk of rapid translation into physical self-injury, serving as a critical red flag for early risk assessment during the initial hospital presentation. Similarly, the peak in neurological-like and somatic symptoms suggests that physical complaints frequently serve as the primary channel for unexpressed psychological trauma in this population. This clinical shift directly justifies the observed pharmacological trends, where the higher rate of treatment initiation and the preferential use of atypical antipsychotics and benzodiazepines over antidepressants were clinically dictated by the need for rapid behavioral stabilization and safe management of acute psychomotor agitation during inpatient stay.

## 5. Limitations

This study has several limitations that should be acknowledged. First, it is a single-center study, which may limit the generalizability of the findings to other healthcare settings or contexts beyond those analyzed, and its retrospective design limits causal inferences and may be subject to incomplete or inaccurate data recording. Furthermore, the sample was exclusively drawn from patients admitted to our unit, excluding those who declined hospitalization. This may have introduced selection bias, as these patients might exhibit clinical or demographic characteristics different from those of the studied population. Additionally, our analysis could not control for potential shifts in referral pathways from primary care pediatricians or the progressive increase in public and family awareness regarding youth mental health during the pandemic years, which may have artificially increased service utilization independently of clinical severity.

Despite using standardized criteria, potential variability in diagnostic interpretation could have influenced the categorization of patients. Additionally, due to the retrospective design, researchers were not blinded to the pandemic periods during data extraction, and inter-rater reliability was not formally assessed; however, any ambiguities were systematically resolved through consensus with senior neuropsychiatrists. The exclusive use of categorical variables constrains the evaluation of more complex or quantitative relationships. The different chronological durations of the three tracking windows represent an inherent limitation of this retrospective design, which was methodologically addressed by applying monthly normalized admission rates to ensure reliable clinical comparisons. These factors should be considered when interpreting the results, highlighting the need for future studies with larger populations and standardized quantitative data collection, enabling more complex statistical analyses and providing a deeper understanding of the long-term effects of the COVID-19 pandemic.

Furthermore, a key limitation of this study is its uncontrolled, retrospective, single-center design, which precludes the establishment of definitive causal relationships. The observed fluctuations in psychiatric presentations cannot be attributed solely to the pandemic or related lockdowns. It is crucial to acknowledge that secular trends in child and adolescent mental health—specifically regarding the rising incidence of anxiety, depressive symptoms, and self-harm—were already shifting globally in the years leading up to 2020 [90,91,92]. Consequently, the pandemic may have acted as an exacerbating factor or a temporal catalyst rather than the exclusive root cause of these clinical patterns. Future multi-center prospective studies are needed to better disentangle pandemic-specific effects from these ongoing, long-term societal trends.

## 6. Conclusions

In conclusion, our study documents significant shifts in the psychiatric phenotypes of child and adolescent admissions across the pre-COVID-19, COVID-19, and post-COVID-19 eras, reflecting an increasing complexity in the presentations to our unit. Beyond documenting changes in the frequency of specific disorders, our findings suggest a transition toward more multifaceted clinical profiles characterized by the coexistence of affective symptoms, self-harm, suicidality, eating disorders, and functional neurological-like symptoms. The persistence of these patterns after the end of pandemic restrictions indicates that these changes extended beyond the acute emergency phase and continued to influence the case-mix of children and adolescents requiring hospitalization.

Rather than proving a direct causal impact, these findings demonstrate a clear temporal association between the pandemic timeline and the emergence of these complex profiles, where physical complaints frequently serve as a somatic channel for psychological distress and mood disturbances carry a heightened risk of rapid translation into physical self-injury. Consequently, these emerging patterns highlight the critical importance of recognizing overlapping symptom profiles early in the clinical pathway. Child and adolescent mental health services must transition from traditional, single-diagnosis frameworks toward integrated care networks. Practically, strengthening community-based early intervention and creating rapid-access crisis support are essential steps to manage severe emotional dysregulation at a community level, thereby preventing emergency department overcrowding and reducing the need for acute psychiatric hospitalization. Future multi-center prospective studies are needed to confirm these trends and further examine the underlying mechanisms of this emerging phenotypic complexity.

## Figures and Tables

**Table 1:** Patient demographics and hospitalization data: pre-COVID-19, COVID-19, and post-COVID-19 groups.

		Pre-COVID-19 Group (12 Months)	COVID-19 Group (16 Months)	Post-COVID-19 Group (13 Months)	Statistical Test	*p*-Value
Patients (M:F)		112 (57:55)	231 (116:115)	162 (79:83)	χ^2^ = 0.54	0.763
Mean age ± SD		11.0 ± 3.9 years	11.2 ± 4.3 years	10.2 ± 4.6 years	*H* = 2.45	0.294
Mean duration ± SD of 1st hospitalization (range)		8.4 ± 8.0 days (2–51 days)	7.9 ± 8.1 days (2–91 days)	6.8 ± 8.5 days (1–60 days)	*H* = 1.34	0.512
Average Monthly Admission Rate (patients/month)		9.33	14.44	12.46	-	-
Patients readmitted in the same period		8 (7.2%)	27 (11.5%)	10 (6.1%)	χ^2^ = 3.81	0.149
Mean duration ± SD of rehospitalization (*n* of patients)	2nd	5.0 ± 4.1 days (8)	7.0 ± 3.5 days (27)	14.8 ± 9.5 days (10)	*H* = 5.22	0.073
	3rd	7 days (1)	9.7 ± 5.0 days (9)	61 days (1)	-	-
	4th	-	16.8 ± 20.3 days (6)	-	-	-
	5th	-	21 days (1)	-	-	-
Percentage of admissions through ER		36.4%	51.1%	28.3%	χ^2^ = 18.24	**<0.001**
Percentage of admissions scheduled due to worsening of symptoms		5.8%	7.9%	15%	χ^2^ = 7.78	**0.020**

Legend: ER = emergency room; F = females; *H* = Kruskal–Wallis; M = males; *n* = number; SD = standard deviation; χ^2^ = chi-square.

**Table 2:** Pharmacological treatments across pre-COVID-19, COVID-19, and post-COVID-19 groups.

Pharmacological Variables	Pre-COVID-19 Group (*N* = 112) *n* (%)	COVID-19 Group (*N* = 231) *n* (%)	Post-COVID-19 Group (*N* = 162) *n* (%)
**Pre-admission Treatment**	34 (30.4%)	109 (47.2%)	65 (40.1%)
Antipsychotics	25 (20.7%)	78 (28.1%)	50 (28.9%)
Mood stabilizers	16 (13.2%)	42 (15.1%)	34 (19.7%)
Benzodiazepines	16 (13.2%)	32 (11.5%)	25 (14.5%)
Antidepressants	2 (1.7%)	23 (8.4%)	9 (5.2%)
Psychostimulants	0 (0.0%)	1 (0.4%)	4 (2.3%)
**In-hospital Management**			
Started a new therapy	40 (33.1%)	132 (47.5%)	82 (47.4%)
Increased dosage	11 (9.1%)	16 (5.8%)	10 (5.8%)
Switched pharmacological class	7 (5.8%)	32 (11.5%)	28 (16.2%)
Most Used Drugs During Hospitalization *			
Antipsychotics	45 (37.2%)	132 (47.5%)	82 (47.4%)
Benzodiazepines	23 (19.0%)	82 (29.5%)	59 (34.1%)
Mood stabilizers	25 (20.7%)	84 (30.1%)	51 (29.5%)
Antidepressants	15 (12.4%)	28 (10.1%)	12 (6.9%)
Psychostimulants	2 (1.7%)	8 (2.9%)	6 (3.5%)

Note: Percentages and raw frequencies in this table reflect the total longitudinal hospitalizations and treatment courses managed across the periods (N Pre-COVID-19 = 121, N COVID-19 = 278, N Post-COVID-19 = 173), while the column headers represent the unique patient population (*n* = 112, *n* = 231, *n* = 162) after removing readmission redundancies for demographic tracking. Due to combination therapies and multiple clinical admissions, the sum of pharmacological categories may exceed the number of individual patients.

## Data Availability

The data presented in this study are available on request from the corresponding author. The data are not publicly available due to privacy and ethical reasons.

## Supplementary Materials

The following supporting information can be downloaded at https://www.mdpi.com/article/10.3390/children13070926/s1, Table S1. Correlation Matrix-Pre-COVID Period (N = 121). Table S2. Correlation Matrix-COVID Period (N = 231). Table S3. Correlation Matrix-Post-COVID Period (N = 162).
