# Supplementary material for: Impact of the COVID-19 Pandemic on Pediatric Neuropsychiatric Disorders: A Retrospective Study at a Single Center for Child Neurology and Psychiatry
Source: Children (Basel). 2026 Jul 14;13(7):926. doi: 10.3390/children13070926 (PMC13407024; doi:10.3390/children13070926)
Supplement: Supplementary file 1 [file children-13-00926-s001.zip › children-4378981-supplementary.pdf]

Supplementary Tables S1-S3: Correlation Matrices

Notes on table reading: Each cell presents the Pearson correlation coefficient (*r*) and its respective exact *p-value* calculated based on the specific sample sizes across the three periods.

Legend: A: anxiety; H: headache; MD: mood disturbance; ND: nutritional disorder; NS: neurological-like symptoms; PA: psychomotor agi-tation; S: suicidality; SH: self-harm.

Table S1. Correlation Matrix - Pre-COVID Period (N = 121)

Variables	PA	S	SH	MD	ND	A	H	NS
**PA**	1							
**S**	r = -0.002 p = 0.986	1						
**SH**	r = 0.180 p = 0.048	r = 0.270 p = 0.003	1					
**MD**	r = 0.360 p = <0.001	r = 0.017 p = 0.853	r = 0.073 p = 0.427	1				
**ND**	r = -0.230 p = 0.011	r = -0.095 p = 0.301	r = -0.110 p = 0.230	r = -0.068 p = 0.459	1			
**A**	r = 0.057 p = 0.535	r = 0.049 p = 0.594	r = 0.047 p = 0.609	r = -0.086 p = 0.349	r = 0.031 p = 0.736	1		
**H**	r = -0.260 p = 0.004	r = -0.120 p = 0.190	r = -0.150 p = 0.101	r = -0.140 p = 0.126	r = -0.130 p = 0.156	r = 0.004 p = 0.968	1	
**NS**	r = -0.220 p = 0.015	r = -0.150 p = 0.101	r = -0.180 p = 0.048	r = -0.220 p = 0.015	r = -0.160 p = 0.080	r = -0.170 p = 0.063	r = -0.022 p = 0.811	1

Table S2. Correlation Matrix - COVID Period (N = 231)

Variables	PA	S	SH	MD	ND	A	H	NS
**PA**	1							

**S**	r = 0.038<br>p = 0.528	1						
**SH**	r = 0.120<br>p = 0.046	r = 0.370<br>p = <0.001	1					
**MD**	r = 0.130<br>p = 0.030	r = 0.030<br>p = 0.618	r = 0.230<br>p = <0.001	1				
**ND**	r = -0.170<br>p = 0.004	r = -0.055<br>p = 0.361	r = -0.033<br>p = 0.584	r = 0.200<br>p = <0.001	1			
**A**	r = -0.088<br>p = 0.143	r = -0.036<br>p = 0.550	r = -0.037<br>p = 0.539	r = -0.047<br>p = 0.435	r = -0.037<br>p = 0.539	1		
**H**	r = -0.240<br>p = <0.001	r = -0.160<br>p = 0.007	r = -0.130<br>p = 0.031	r = -0.210<br>p = <0.001	r = -0.130<br>p = 0.031	r = -0.033<br>p = 0.584	1	
**NS**	r = -0.190<br>p = 0.001	r = -0.330<br>p = <0.001	r = -0.230<br>p = <0.001	r = -0.340<br>p = <0.001	r = -0.280<br>p = <0.001	r = -0.071<br>p = 0.238	r = 0.190<br>p = 0.001	1

Table S3. Correlation Matrix - Post-COVID Period (N = 162)

Variables	PA	S	SH	MD	ND	A	H	NS
**PA**	1							
**S**	r = 0.050<br>p = 0.513	1						
**SH**	r = 0.140<br>p = 0.066	r = 0.410<br>p = <0.001	1					
**MD**	r = 0.160<br>p = 0.036	r = 0.310<br>p = <0.001	r = 0.650<br>p = <0.001	1				
**ND**	r = -0.140<br>p = 0.066	r = -0.072<br>p = 0.347	r = -0.006<br>p = 0.940	r = 0.160<br>p = 0.036	1			
**A**	r = -0.190<br>p = 0.012	r = -0.097<br>p = 0.204	r = -0.140<br>p = 0.066	r = -0.170<br>p = 0.026	r = -0.085<br>p = 0.266	1		
**H**	r = -	r = -	r = -	r = -	r = -	r = -	1	

	0.065 p = 0.395	0.023 p = 0.764	0.033 p = 0.667	0.041 p = 0.592	0.033 p = 0.667	0.025 p = 0.744		
**NS**	r = - 0.130 p = 0.088	r = - 0.280 p = <0.001	r = - 0.360 p = <0.001	r = - 0.460 p = <0.001	r = - 0.400 p = <0.001	r = - 0.095 p = 0.214	r = - 0.071 p = 0.353	1
